# Variability in Reporting eGFR at Dialysis Initiation in Canada: A Research Letter

**DOI:** 10.1177/20543581231203065

**Published:** 2023-09-29

**Authors:** Michael Chiu, Samuel A. Silver, Laura Berall, Isabelle Ethier, Claire Harris, Keigan More, Annie-Claire Nadeau-Fredette, Karthik Tennankore, Jay Hingwala

**Affiliations:** 1 Division of Nephrology, Department of Medicine, London Health Sciences Centre, Western University, London, ON, Canada; 2 Division of Nephrology, Kingston Health Sciences Center, Queen’s University, Kingston, ON, Canada; 3 Division of Nephrology, Department of Medicine, Humber River Hospital, Queen’s University, Toronto, ON, Canada; 4 Division of Nephrology, Department of Medicine, Centre Hospitalier de l’Université de Montréal, Montréal, QC, Canada; 5 Health Innovation and Evaluation Hub, Centre de recherche du Centre Hospitalier de l’Université de Montréal, Montréal, QC, Canada; 6 Division of Nephrology, Department of Medicine, Vancouver General Hospital, The University of British Columbia, Vancouver, BC, Canada; 7 Division of Nephrology, Department of Medicine, Dalhousie University, Halifax, NS, Canada; 8 Division of Nephrology, Department of Medicine, Hôpital Maisonneuve-Rosemont, Université de Montréal, Montreal, QC, Canada; 9 Division of Nephrology, Winnipeg Health Sciences Centre, University of Manitoba, Winnipeg, MB, Canada

**Keywords:** dialysis, quality improvement, renal quality indicators

## Abstract

**Background:**

Estimated glomerular filtration rate (eGFR) at dialysis initiation is increasingly recognized as a key quality indicator (QI) for patients with end-stage kidney disease (ESKD). Specifically, guidelines recommend assessing deferral of dialysis initiation until symptoms arise or if the eGFR is ≤6 mL/min/1.73 m^2^. Despite the recognition of the importance of this QI, how eGFR at the time of dialysis initiation is defined, collected, and tracked at dialysis centers across Canada remains unknown.

**Objectives:**

To identify how provincial renal programs define eGFR at dialysis initiation, to compare practice across Canadian provinces, and to determine if there is a consistent benchmark for deferred dialysis start.

**Design:**

Cross-sectional survey distributed to the medical leads of each provincial renal program, administered from July 2021 to November 2021. Quebec was not included given it did not yet participate in Canadian Organ Replacement Register (CORR) data submission.

**Setting:**

The survey was designed and distributed by the Canadian Society of Nephrology Quality Improvement & Implementation Science Committee (CSN-QUIS) Indicator Working Group.

**Methods:**

The survey asked respondents on how eGFR is defined, collected, reported, and perceived barriers to QI data collection. The National Senior Renal Leaders Forum helped identify the key provincial medical leads to disseminate the survey for completion.

**Results:**

Surveys were distributed to the medical leads of the 9 provincial renal programs that participate in CORR. In total, there were 8 responses. Five provinces submit eGFR for all new dialysis starts and 3 provinces only submit this information for chronic patients. There is variation in determining when a patient with acute kidney injury requiring dialysis is classified as a chronic patient. Four provinces use a 30-day trigger, 3 provinces use a 90-day trigger, and the patient’s nephrologist makes this determination in 1 province. The creatinine used for the eGFR at dialysis initiation was the value measured on the first dialysis session (ie, day 0) for 5 provinces; the last outpatient clinic creatinine value in 2 provinces, and 1 province did not have a standard definition. Three provinces did not have a benchmark target for eGFR at dialysis initiation, 1 province had a target of <9.5 mL/min/1.73 m^2^, 3 provinces had a target of <10 mL/min/1.73 m^2^, 1 province had a target of <15 mL/min/1.73 m^2^. All 8 responding provincial medical leads support the establishment of a national benchmark for this measure.

**Limitations:**

This survey was restricted to provincial medical leads and therefore is unable to determine practice at individual dialysis sites. The survey was not anonymous, so it may be subject to conformity bias.

**Conclusions:**

There is wide variability in how eGFR at dialysis initiation is measured and reported across Canada. Additionally, there is no consensus on a benchmark target for an intent-to-defer dialysis strategy. Standardization of target eGFR at dialysis initiation may facilitate national reporting and quality improvement initiatives.

## Abrégé

**Contexte:**

Le débit de filtration glomérulaire estimé (DFGe) à l’amorce de la dialyse est de plus en plus reconnu comme un indicateur clé de la qualité (IQ) chez les patients atteints d’insuffisance rénale terminale (IRT). Plus précisément, les lignes directrices recommandent d’évaluer la possibilité de reporter l’initiation de la dialyse jusqu’à l’apparition des symptômes ou l’atteinte d’un DFGe égal ou inférieur à 6 ml/min/1,73 m^2^. Bien qu’on reconnaisse l’importance de cet IQ, on ignore encore comment le DFGe est défini, mesuré et suivi au moment de l’initiation de la dialyse dans les centres de dialyse canadiens.

**Objectifs:**

Déterminer la façon dont les programmes rénaux provinciaux définissent le DFGe à l’initiation de la dialyse, comparer les pratiques en cours dans les différentes provinces canadiennes et déterminer s’il existe une cible de référence commune pour une initiation différée de la dialyse.

**Conception:**

Un sondage transversal distribué entre juillet et novembre 2021 aux directeurs médicaux de chaque programme provincial de soins rénaux. Le Québec n’a pas été inclus puisque la province n’a pas encore participé au Registre canadien des insuffisances et des transplantations d’organes (RCITO).

**Cadre:**

Le sondage a été conçu et distribué par le Groupe de travail sur les indicateurs du Quality Improvement & Implementation Science Committee de la Société canadienne de néphrologie (CSN-QUIS).

**Méthodologie:**

Les répondants au sondage devaient décrire la façon dont le DFGe est défini, mesuré et rapporté, ainsi que les obstacles perçus à la collecte de données sur les IQ. Le sondage a été distribué aux directeurs médicaux provinciaux identifiés par le biais du National Senior Renal leaders Forum.

**Résultats:**

Le sondage a été distribué aux directeurs médicaux des neuf programmes provinciaux de soins rénaux participant au RCITO; huit ont répondu. Cinq provinces soumettent le DFGe pour toute nouvelle initiation d’un traitement de dialyse; trois provinces ne soumettent cette information que pour les patients atteints d’insuffisance rénale chronique. Il existe des différences entre les provinces dans la détermination du moment où un patient passe de l’insuffisance rénale aiguë nécessitant une dialyse à l’insuffisance rénale chronique. Quatre provinces utilisent un délai de 30 jours, trois provinces utilisent un délai de 90 jours et dans la dernière province, cette détermination est faite par le néphrologue du patient. Dans cinq des huit provinces sondées, le taux de créatinine utilisé pour établir le DFGe à l’initiation de la dialyse est la valeur mesurée à la première séance de dialyse (au jour 0); deux provinces utilisent la valeur de créatinine mesurée lors de la dernière visite en ambulatoire, et une province n’a pas de définition normalisée. Trois provinces n’ont pas de cible de référence pour le DFGe à l’initiation de la dialyse; cette cible est de moins de 9,5 ml/min/1,73 m^2^ dans une province, de moins de 10 ml/min/1,73 m^2^ dans trois provinces, et de moins de 15 ml/min/1,73 m^2^ dans une province. Les huit responsables médicaux provinciaux ayant répondu au sondage appuient l’établissement d’une valeur de référence nationale pour cette mesure.

**Limites:**

Ce sondage n’a été envoyé qu’aux directions médicales provinciales, par conséquent, il ne permet pas de déterminer les pratiques en cours dans chaque site de dialyse. Le sondage n’étant pas anonyme, il pourrait comporter un biais de conformité.

**Conclusion:**

Il existe une grande variabilité au Canada dans la façon dont le DFGe est mesuré et rapporté au début de la dialyse. On observe en outre une absence de consensus quant à une cible de référence pour une stratégie d’initiation différée de la dialyse. La normalisation de la valeur cible de DFGe au début de la dialyse pourrait faciliter les initiatives nationales de déclaration et d’amélioration de la qualité.

## Introduction

The incidence and prevalence of end-stage kidney disease (ESKD) is rising. According to the most recent Canadian Organ Replacement Register (CORR) report, the incidence of ESKD patients increased by 24% between 2012 and 2021. Currently, there are more than 29 000 people receiving dialysis in Canada.^1^

The optimal time to start dialysis has evolved, with a pivotal change after the publication of the Initiating Dialysis Early and Late (IDEAL) trial in 2010. Prior to the IDEAL trial, the tendency to start dialysis earlier was based on the perception that it maintained cognition, optimized quality of life, and improved survival.^2^ However, the IDEAL study demonstrated that starting dialysis early (eGFR of 10-14 mL/min/1.73 m^2^) compared to late (eGFR 5-7 mL/min/1.73 m^2^), was not associated with improved survival, cardiovascular events, or infections.^3^ Not only were patients in the late start cohort deferring dialysis by approximately 6 more months, but a subsequent analysis demonstrated that there was no difference in quality of life but increased dialysis costs for patients who started dialysis early.^4^ A more recent large observational cohort study investigated the mortality risk of starting dialysis at a higher eGFR. It found that very early dialysis starts (eGFR 14-15 mL/min/1.73 m^2^) resulted in decreased mortality. However, these patients started dialysis, on average, 4 years earlier, which may not outweigh the marginal mortality benefit.^5^ The IDEAL study led to a shift in practice and updated guidelines, where dialysis is deferred until uremic symptoms develop or until eGFR reaches ≤6 mL/min/1.73 m^2^.^6^ In Canada, between 2006 and 2015, the proportion of early dialysis starts (starting with an eGFR >10.5 mL/min/1.73 m^2^) decreased from 39% prior to the IDEAL trial to 34% after publication of the results.^7^

Despite this improvement, the most recently published data reveal that 34% of patients still start dialysis early. This is suboptimal as there are many patients who may start dialysis too early, and it is unknown how this varies across provinces. Thus, the importance of developing a Canada-wide quality indicator (QI) that ensures uniform care and informs efforts to improve the optimal timing to start dialysis is evident.

Imperative to the intent-to-defer strategy is awareness of how eGFR is reported at dialysis initiation across Canada. Comparing eGFR at dialysis initiation is possible with database information that is already housed and reported by the Canadian Institute for Health Information and CORR. CORR is a pan-Canadian database that receives data from provincial renal programs, excluding Quebec. It provides robust statistics on Canadian dialysis activity. To our knowledge, no prior study has explored if eGFR is uniformly defined and collected, which makes it difficult to compare performance across Canadian provinces.

## Methods

This was a cross-sectional survey of the provincial renal programs across Canada that submitted data to CORR. Quebec was excluded since it has not yet fully participated with CORR. The survey was created by an author, with multiple iterations of pilot testing by 2 other members of the Canadian Society of Nephrology Quality Improvement & Implementation Science Committee (CSN-QUIS) for questions to include content expertise, comprehension, and ambiguity. After consensus was achieved, the survey was conducted from July to November 2021. Survey respondents were from the National Senior Renal Leaders Network or their delegates. This network includes senior executives from adult nephrology programs in Canada.^8^ The survey was created to identify how each province reports clinical data for each new dialysis start (Supplement 1). Participation was voluntary and without incentivization. The survey explored themes related to eGFR at dialysis initiation, including measurement, data collection, reporting, and monitoring. The responses were submitted to the CSN-QUIS and summarized by the authors. This survey was deemed exempt from ethics review, based on TCPS 2 Article 2.5, by the Institutional Review Board at Queen’s University.

## Results

Survey responses were received from 8/9 provinces.

### Data Submission

Seven provinces report creatinine to calculate eGFR at dialysis start, and 1 reports both creatinine and eGFR. Five provinces report data for all new dialysis starts (both acute and chronic), whereas 3 provinces only report data for chronic patients. For acute kidney injury requiring dialysis, the timing to determine if the patient is chronic also varies. Four provinces use a 30-day trigger, 3 provinces use a 90-day trigger, and the physician makes this determination in 1 province. The creatinine used to calculate eGFR at dialysis initiation is also inconsistent. Five provinces use the creatinine on the day of dialysis initiation (day 0), 2 provinces use the most recent outpatient creatinine prior to dialysis start, and 1 province does not have a standard practice and sometimes uses the creatine 1 week after dialysis.

### Target eGFR

One province had a target of <9.5 mL/min/1.73 m^2^, 3 provinces had a target of <10 mL/min/1.73 m^2^, 1 province had a target of <15 mL/min/1.73 m^2^, and 3 provinces did not have a target. All responding provinces wanted a national benchmark to be established. Moreover, all provinces were interested in participating in a project to standardize creatinine and eGFR reporting at dialysis initiation.

## Discussion

Variability exists in how data is collected and reported for dialysis starts in Canada. There are discrepancies in which creatinine value is reported to CORR, and there is room for error if creatinine values are collected after dialysis initiation, which occurs in 1 province. Furthermore, it is inconsistent when a patient is determined to be dialysis dependent. Importantly, there are no consensus targets for dialysis initiation. These discrepancies make it difficult to compare the performance across the country. What is reassuring is that there is a desire among all programs to develop a national benchmark to help improve performance.

One solution to the variability in the measurement of eGFR at dialysis start is to create standard work. Standard work is a quality improvement tool that creates precise procedures to minimize variability and optimize efficiency.^9^ Standard work allows for consistent measurements of process measures that can be used to drive performance across jurisdictions and allow for continuous quality improvement.^10^

Our group suggests that standard work can be applied to this process (Table 1). First, a standard definition of eGFR at dialysis initiation is required. Additionally, there must be a standard definition of what constitutes a chronic dialysis patient. Subsequently, eGFR reporting can be divided into 5 specific tasks (Table 1). Data collection should be delegated to specific data collector(s). The frequency of collection should be done at least monthly. Each site then submits the information to CORR at a maximum frequency of every 6 months (this is currently the quickest possible reporting by CORR). CORR then pools and analyzes the data every 6 to 12 months. This data can be aggregated at the provincial level and reported with the benchmark and national averages. The data is then sent to the provincial medical leads every 6 to 12 months in the form of a scorecard (Supplement 2). Provincial leads then disseminate the information to each site. Finally, based on the standard reports, each site conducts local quality improvement initiatives to improve performance. While the frequency of data collection and reporting may be intermittent, this framework allows for constant feedback and accountability to each site, which is imperative for continuous quality improvement and optimal patient outcomes.

**Table 1.** Example of Standard Work for eGFR Reporting.

Task no.	Task/activity in Plan-Do-Study-Act (PDSA) cycle	Frequency/timeline	Who performs	Other points
1	Data collector collects new dialysis starts for Canadian Organ Replacement Register (CORR) data	Site specific (max = monthly)	Data collector (electronic medical record, dedicated data collector)	
2	Data submitted to CORR	Site specific (max = every 6 months)	Data collector/Clerk	
3	CORR pools & analyzes data	Every July 1 and January 1 (every 6-12 months)	CORR	Done at provincial level with national averages and benchmarks reported
4	Report sent to provincial medical and administrative leads	August 1 and February 1 (every 6 months)	CORR	
5	Provincial leads disseminate information locally	Every 6 months	Provincial leads	
6	Local quality improvement initiatives	Implementation (PDSA) cycles every 4-6 months	Local quality improvement leads	

*Note.* eGFR = estimated glomerular filtration rate.

It is also clear that a national benchmark for eGFR at dialysis initiation is needed. The most common provincial eGFR to initiate dialysis is <10 mL/min/1.73 m^2^, despite the CSN guidelines suggesting a target of <6 mL/min/1.73 m^2^ is acceptable without clinical symptoms.^6^ Further, a national consensus among provincial medical leaders to determine the most appropriate benchmark for achieving the intent-to-defer strategy is an important next step. This benchmark will need to allow flexibility in clinical decision making, as a target of 100% of all dialysis starts is not appropriate, given some patients may require dialysis at higher eGFRs for other indications. Furthermore, the clinical indication for dialysis initiation (eg, refractory volume overload), especially in patients who may start dialysis earlier, should be reported.

This is the first survey to demonstrate the highly variable practice across Canada in regards to QI measurement, with a focus on delayed dialysis start. There was 89% engagement across Canada, which suggests interest in improving the eGFR at dialysis initiation renal quality indicator. This study has limitations. First, this is a small survey of provincial medical leads. Since practice may be variable within each province, it may not have captured the typical practice at each site level. However, it still highlights the need to create standard practice across renal programs in Canada. Second, this survey was not anonymous, which may create conformity bias in that respondents may feel obliged to provide more positive responses. However, given the diversity of responses, we do not think this significantly affected our results.

## Conclusion

There is significant variation in reporting eGFR at dialysis initiation. The suggested framework standardizes data collection and reporting to CORR, which promotes feedback to provincial programs that will drive quality improvement initiatives. This report serves as a call to action to standardize targets for eGFR at dialysis initiation and to create a national benchmark that all programs can strive for.

## Supplemental Material

sj-docx-1-cjk-10.1177_20543581231203065 – Supplemental material for Variability in Reporting eGFR at Dialysis Initiation in Canada: A Research Letter Click here for additional data file. Supplemental material, sj-docx-1-cjk-10.1177_20543581231203065 for Variability in Reporting eGFR at Dialysis Initiation in Canada: A Research Letter by Michael Chiu, Samuel A. Silver, Laura Berall, Isabelle Ethier, Claire Harris, Keigan More, Annie-Claire Nadeau-Fredette, Karthik Tennankore and Jay Hingwala in Canadian Journal of Kidney Health and Disease
