# Supplementary material for: Variability in Reporting eGFR at Dialysis Initiation in Canada: A Research Letter
Source: Can J Kidney Health Dis. 2023 Sep 29;10:20543581231203065. doi: 10.1177/20543581231203065 (PMC10541730; doi:10.1177/20543581231203065)
Supplement: sj-docx-1-cjk-10.1177_20543581231203065 – Supplemental material for Variability in Reporting eGFR at Dialysis Initiation in Canada: A Research Letter [file sj-docx-1-cjk-10.1177_20543581231203065.docx]

**SUPPLEMENT 1 – Survey**

PROVINCE: __________ CONTACT: ____________ SITE:____________

Standardized template of questions (specific to eGFR at dialysis start)

1. Does your site collect creatinine (Cr) or eGFR at dialysis initiation for

Canadian Organ Replacement Registry (CORR) data submission?

A) creatinine only

B) eGFR only

C) both

1. Who gets eGFR/Cr at dialysis start reported at your site?
2. acute kidney injuries/unplanned starts
3. chronic dialysis patients
4. all
5. other __________________________________________
6. What definition is used at your site for eGFR/Cr at dialysis start?

A) eGFR/Cr on the day of first dialysis prior to initiation

B) eGFR/Cr as outpatient provided within 1 week before dialysis start

C) any eGFR/Cr as outpatient before dialysis start

D) no standardized eGFR/Cr collection date or time

E) other ________________________________________________

1. If you measure eGFR at dialysis start, do you adjust for race?
2. yes, we adjust for black race only
3. yes, we adjust for multiple races
4. no, we do not adjust for races
5. not sure
6. other: ___________________
7. What is your preferred definition of eGFR at dialysis start?

A) eGFR at time of first dialysis session

B) last eGFR as outpatient before dialysis start

C) other ___________________________________________________

1. Who collects eGFR/Cr at dialysis start for CORR submission at your site?
2. dedicated data collectors
3. dialysis nurses/Clinical Resource Nurse (CRN)
4. administrative database collector
5. physician
6. automated electronic data capture from a medical record
7. other ___________________________________________________
8. Once eGFR/Cr is collected, who enters this into CORR?

A) dedicated data collectors

B) dialysis nurses/CRNs

C) administrative database collector

D) physician

1. automated electronic data capture from medical record

F) other ___________________________________________________

1. How often is the eGFR/Cr data captured for CORR?
2. daily (i.e., on day the patient starts dialysis)
3. batching weekly
4. batching monthly
5. other ­­­­­___________________________________
6. How does the process to collect eGFR/Cr at dialysis start vary across your Province? (choose one or more below)

A) no variation

B) the definition is different (ex. some programs use first day of HD and other use last outpatient value)

C) when the data is collected is different (ex. some programs collect data daily for each dialysis start and some batch data collection for each month)

D) when the data is entered into CORR is different (ex. daily, weekly, random, monthly)

E) not sure

F) other _______________________________________

1. With acute kidney injuries (AKIs) – when is CORR registration and data collection triggered?
2. at 90 days
3. when clinician deems patient chronic
4. other _________________________________________________
5. What are some perceived limitations with the current state for collecting eGFR/Cr at dialysis start? (choose one or more below)
6. same equation is not used to calculate eGFR/Cr (MDRD vs CKD-EPI)
7. eGFR/Cr at dialysis start does not have uniform and concise definition
8. lack of adequate resources to collect data
9. other ____________________________
10. Do you have an eGFR/Cr at dialysis start target/benchmark? If so, what is it (please list eGFR if known)?
11. Provincial target ______________
12. site target ___________________
13. guideline based ____________________________ (which guideline)
14. other _________________________________________
15. Which eGFR/Cr at dialysis start benchmark should be established?

A) Provincial target

B) site average

C) National target

D) guideline based ____________ (which guideline)

E) other _________________________________________

1. Are you interested in participating in a project to standardize and report eGFR/Cr at dialysis start at your site?
2. yes
3. no
4. What local projects/unpublished work, if any, has your centre or Province tried related to reporting eGFR/Cr at dialysis start?

_________________________________________________________________________________________

_________________________________________________________________________________________

1. Local champion identified (name, role)?

______________________________

**SUPPLEMENT 2 – Sample Report with hypothetical data**

Key Performance Indicator: Estimated GFR at dialysis initiation

***Definition:*** Percentage of incident chronic dialysis patients starting dialysis with an eGFR ≤ 9.5 mL/min/1.73 m^2^

***Proposed Target:*** >90^th^ percentile

**Provincial Data:** Province 7

|  | **Current Performance (2020)** | | | | **Historical Performance** | | |
| --- | --- | --- | --- | --- | --- | --- | --- |
|  | Actual | Target | National Average | Rank | 2019 | 2018 | 2017 |
| Percent | 50.0 | >79.5 | 68.1 | 8/9 | 51.8 | 56.2 | 49.7 |
| Numerator | 71 |  |  |  | 85 | 82 | 79 |
| Denominator | 142 |  |  |  | 164 | 146 | 159 |

**Provincial Comparison**

| **Province** | **2020** | **2019** | **2018** | **2017** |
| --- | --- | --- | --- | --- |
| 1 | 49.0 | 51.9 | 45.9 | 48.1 |
| 2 | 79.4 | 81.9 | 80.7 | 77.4 |
| 3 | 73.1 | 73.3 | 71.6 | 69.2 |
| 4 | 80.0 | 77.4 | 76.5 | 77.0 |
| 5 | 70.4 | 68.4 | 65.4 | 64.2 |
| 6 | 75.7 | 76.8 | 83.5 | 78.6 |
| 7 | 50.0 | 51.8 | 56.2 | 49.7 |
| 8 | 72.0 | 62.5 | 60.0 | 77.5 |
| 9 | 69.9 | 79.6 | 84.2 | 67.3 |
| National Average | 68.1 | 67.8 | 65.3 | 63.6 |

**SUPPLEMENT 3 – Survey Results**

Question 1

Question 2

Question 3

Question 4

Question 5

Question 6

Question 7

Question 8

Question 9

Question 10

Question 11

Question 12

Question 13

Question 14
